# Extraction of Radiological Characteristics From Free-Text Imaging Reports Using Natural Language Processing Among Patients With Ischemic and Hemorrhagic Stroke: Algorithm Development and Validation

**DOI:** 10.2196/42884

**Published:** 2023-06-06

**Authors:** Enshuo Hsu, Abdulaziz T Bako, Thomas Potter, Alan P Pan, Gavin W Britz, Jonika Tannous, Farhaan S Vahidy

**Affiliations:** 1 Center for Health Data Science and Analytics Houston Methodist Research Institute Houston, TX United States; 2 School of Biomedical Informatics University of Texas Health Science Center at Houston Houston, TX United States; 3 Department of Neurosurgery Houston Methodist Neurological Institute Houston, TX United States; 4 Department of Neurology Weill Cornell Medical College New York, NY United States; 5 Department of Population Health Sciences Weill Cornell Medical College New York, NY United States

**Keywords:** natural language processing, deep learning, electronic health records, ischemic stroke, cerebral hemorrhage, neuroimaging, computed tomography, stroke, radiology

## Abstract

**Background:**

Neuroimaging is the gold-standard diagnostic modality for all patients suspected of stroke. However, the unstructured nature of imaging reports remains a major challenge to extracting useful information from electronic health records systems. Despite the increasing adoption of natural language processing (NLP) for radiology reports, information extraction for many stroke imaging features has not been systematically evaluated.

**Objective:**

In this study, we propose an NLP pipeline, which adopts the state-of-the-art ClinicalBERT model with domain-specific pretraining and task-oriented fine-tuning to extract 13 stroke features from head computed tomography imaging notes.

**Methods:**

We used the model to generate structured data sets with information on the presence or absence of common stroke features for 24,924 patients with strokes. We compared the survival characteristics of patients with and without features of severe stroke (eg, midline shift, perihematomal edema, or mass effect) using the Kaplan-Meier curve and log-rank tests.

**Results:**

Pretrained on 82,073 head computed tomography notes with 13.7 million words and fine-tuned on 200 annotated notes, our HeadCT_BERT model achieved an average area under receiver operating characteristic curve of 0.9831, *F*_1_-score of 0.8683, and accuracy of 97%. Among patients with acute ischemic stroke, admissions with any severe stroke feature in initial imaging notes were associated with a lower probability of survival (*P*<.001).

**Conclusions:**

Our proposed NLP pipeline achieved high performance and has the potential to improve medical research and patient safety.

## Introduction

### Overview

Computed tomography (CT) and magnetic resonance imaging (MRI) are the gold standards for assessing and triaging patients with suspected strokes. However, free-text imaging reports containing important radiological findings are embedded in electronic health records (EHRs) systems in an unstructured narrative format, precluding data encoding [1] to enable clinical decisions and support research applications [2-4]. Fortunately, the limitations of unstructured data have been mitigated by recent advancements in information extraction and processing methods, such as natural language processing (NLP).

Traditional rule-based NLP algorithms that use handcrafted dictionaries, keywords, and decision rules to analyze the structure of the language have classically been adopted for analyses of textual data [5-7]. However, the creation and maintenance of decision rules are labor-intensive tasks, and the quality of rules significantly influences model performance. In recent years, data-driven methods, including machine learning and deep learning, have been developed. Machine learning approaches use derived features (eg, term frequency and n-gram) from text to train supervised-learning models (eg, support vector machine [SVM] or random forest) and predict desirable outputs on new documents [3,8,9]. Deep learning methods often involve more sophisticated architectures (eg, recurrent neural networks, convolutional neural networks, and self-attention) and use word embeddings to account for the sequence and context of natural language [1,10,11].

The Bidirectional Encoder Representations from Transformers (BERT) NLP model, which uses a 24-layered deep learning architecture, was published in 2018 and achieved state-of-the-art performance on NLP benchmarks [12]. A clinical version, ClinicalBERT, was later developed by pretraining the BERT model on EHR notes to achieve improved performance on clinical data [13]. Furthermore, the ClinicalBERT model has also been trained and validated for the extraction of radiological features from chest and bone x-ray notes [14,15].

In the context of cerebrovascular disease and stroke, NLP has been applied to classify various stroke phenotypes [3,8,9] and perform feature extraction [1,5,6]. Despite these emerging applications, optimal use of NLP pipelines for stroke research is yet to be achieved. More specifically, limited studies have used BERT to extract important neuroimaging findings, such as midline shift [16] and mass effect [17]. Therefore, the use of NLP-based extraction of many critically important neuroimaging features has not been systematically implemented. We evaluated a deep learning–based NLP model (HeadCT_BERT) that is built upon ClinicalBERT and fine-tuned for the extraction and structured data generation of 13 critical stroke neuroimaging features.

### Related Work

#### NLP on Stroke Imaging Notes

NLP has been adopted to automate stroke acuity classification. Li et al [8] used head CT and MRI radiology reports to train a random forest model for ischemic stroke acuity classification. Kim et al [9] evaluated logistic regression, naïve Bayesian, decision tree, and SVM models to identify ischemic stroke from MRI reports. In addition, Garg et al [3] trained a variety of machine learning algorithms (ie, k-nearest neighbors, SVM, random forest, extra trees classifier, and XGBoost) to identify ischemic stroke subtypes from neurology progress notes and neuroradiology reports. In addition to NLP-based classification algorithms, a few studies adopted NLP for stroke imaging feature extraction. Yu et al [5] used a rule-based NLP tool, CHARTextract, to extract the type of occlusion, presence of established ischemia, and hemorrhage from CT reports. Gordon et al [17] proposed a machine learning–based method using XGBoost to extract the intracranial mass effect. However, there are several untapped avenues for the applications of state-of-the-art NLP methods in the stroke and cerebrovascular disease domain.

#### Fine-Tuning BERT for Medical Imaging Findings Extraction

The most common application of BERT is to fine-tune the out-of-box network for the NLP task. Olthof et al [18] fine-tuned the BERT model with 3268 labeled radiology reports of injured extremities and chest radiographs for extracting the presence of injury. The BERT network was appended with a binary classifier layer and trained (“fine-tuned”) with the labeled reports. The authors reported that BERT outperformed rule-based classifiers and machine learning classifiers and achieved an *F*_1_-score of 0.95 and an area under receiver operating characteristic curve (AUROC) of 0.99. Fink et al [19] fine-tuned the German-language BERT with structured oncology reports for rapid tumor response category classification. The results showed that the BERT model (*F*_1_=0.70) achieved a similar performance as that of medical students (*F*_1_≈0.73), although it was inferior to radiologists’ performance (*F*_1_=0.79).

#### Pretraining and Fine-Tuning BERT for Medical Imaging Findings Extraction

Pretraining BERT with domain-specific text is an additional step that may boost model performance in subsequent fine-tuning. Smit et al [14] used an automatic labeling algorithm to tag 200,000 radiology reports for pretraining. After pretraining, 1000 reports were randomly sampled and annotated by radiologists for fine-tuning. The final NLP model, CheXbert, achieved state-of-the-art performance on one of the largest chest x-ray data sets, MIMIC-CXR, with an *F*_1_-score of 0.798, which is close to radiologists’ performances (*F*_1_=0.805). Dai et al [15] took a similar approach using x-ray radiology reports for bone fracture. The authors developed a rule-based automatic labeling algorithm to label 6048 reports for model pretraining. Subsequently, the model was fine-tuned with a subset of 4890 manually annotated reports for fracture status detection (ie, positive, negative, or uncertain) and fracture type, bone type, and location extraction. To our knowledge, BERT pretraining in the biomedical field is underused and has not been attempted within the cerebrovascular disease domain.

## Methods

### Data Source and Variables

Registry for Neurological Endpoint Assessments among Patients with Ischemic and Hemorrhagic Stroke (REINAH) [20] is a data warehouse built upon the EHR at Houston Methodist, a tertiary health care system serving the greater Houston metropolitan area. REINAH hosts data for over 45,000 patients with cerebrovascular disease, representing over 982,000 neuroimaging records obtained between September 2007 and August 2022. From REINAH, we queried records that (1) had final results available before data collection on July 19, 2021; (2) had an imaging type of “CT head without contrast”; and (3) had attached imaging notes. All imaging notes were written in short paragraphs and stored as plain text. The age, sex, race, ethnicity, BMI, insurance type, stroke type, and National Institutes of Health Stroke Scale scores were extracted from each patient’s initial stroke encounter.

### Ethics Approval

This study was approved by the Houston Methodist Institutional Review Board (PRO00025034).

### Annotation

**Figure 1:**
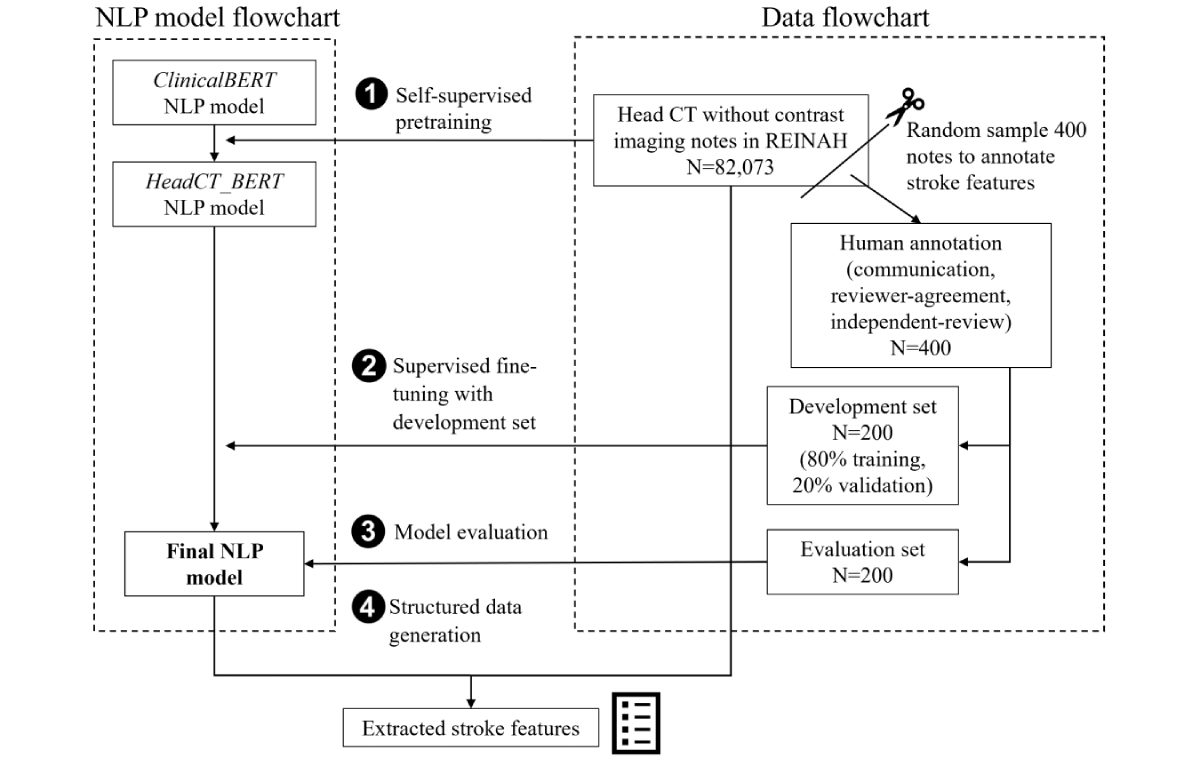
Methodology flowchart. We used unannotated computed tomography (CT) imaging notes to pretrain the natural language processing (NLP) model and used a subset of annotated imaging notes to fine-tune and evaluate it. BERT: bidirectional encoder representations from transformers; REINAH: Registry for Neurological Endpoint Assessments among Patients with Ischemic and Hemorrhagic Stroke.

We identified 20 clinically relevant stroke-related features to extract, including hemorrhage volume, midline shift, herniation, perihematomal edema, white matter hyperintensity, intracerebral hemorrhage (ICH) location, lacunes, old stroke, remote stroke, subacute infarct, cerebral atrophy, intraventricular hemorrhage, acute ischemia, subdural hematoma, subarachnoid hemorrhage, extra-axial hemorrhage, encephalomalacia, mass effect, and location for any non-ICH lesion (finding location). Each imaging note could include none, one, or multiple concepts. As illustrated in Figure 1, we randomly sampled 400 notes for model fine-tuning and evaluation and adopted the Begin-Inside-Outside method [21], which tags the starting position and end position of predetermined imaging features of interest in the text. We then randomly partitioned the 400 samples into the following three data sets: (1) a communication set containing 50 notes; (2) a reviewer-agreement set with 50 notes; and (3) two independent-review sets, each containing 150 notes. Two clinically trained reviewers in neuroimaging (ATB and TP) then manually annotated the imaging notes in 3 sequential stages. In the first stage, the communication set was annotated collaboratively by the 2 reviewers. In the second stage, reviewers performed separate annotations of the reviewer-agreement set, and Kappa statistics and percent agreement were evaluated. Inconsistent annotations were discussed to reach a consensus. Finally, independent review sets were separately annotated. Stroke imaging features that were identified in less than 20 notes were excluded from modeling.

### Text Processing

Before a sequence of human language can be processed by NLP models, the text often goes through processes of segmentation, tokenization, and word embedding [22]. To segment notes, we first fixed a segment length of 32 words and a step size of 10 words. For each note, the first 32 words were taken as a segment, which was then shifted to the right by 1 step (10 words) to isolate the next segment of 32 words. This process was repeated until the end of the note was reached, thereby transforming a single long note into multiple short, overlapping, text segments. For each segment, word tokenization, which transforms sentences and phrases into individual word-tokens, was performed using the WordPiece [23] algorithm implemented in the Python Transformers module (version 4.10.0) and based on a predefined dictionary. In-dictionary words with predetermined tokens (eg, “stroke” and “patient”) were mapped to respective numeric IDs (word embedding). Conversely, out-of-dictionary words (eg, “edema” and “hemorrhage”) were split into multiple in-dictionary tokens and mapped to multiple token IDs (Table 1).

**Table 1:** Examples of text segmentation and word embedding^a^.

Input word	Word-token(s)	Word embedding ID(s)
stroke	stroke	6625
patient	patient	5351
edema	(ed, ##ema)	(5048, 14494)
hemorrhage	(hem, ##or, ##r, ##hage)	(23123, 1766, 1197, 19911)

^a^The WordPiece algorithm takes each word as input. If a word matches a predefined word-token, embedding is done by assigning a token ID to the word. If a word does not match any predefined token, the word is split into multiple fractions and matched with predefined tokens.

### Deep Learning NLP Models

Our NLP model training involved two phases, as follows: (1) an optional general training phase (“pretraining”) that familiarized the model with clinical terminology in head CT notes, and (2) a required task-specific training phase (“fine-tuning”), where the model learned to identify the 13 remaining stroke features (Table S1 in Multimedia Appendix 1).

#### Pretraining

Though NLP models can be trained with solely fine-tuning, recent studies have reported an improved performance after general [12,24] and domain-specific [13,25] pretraining. We used the ClinicalBERT model, which has been pretrained on general English corpora and EHR narratives [13]. We hypothesized that further pretraining it with our head CT notes using masked language model (MLM) [12] would boost the performance for stroke feature extraction. Details of NLP model pretraining are provided in Table S2 in Multimedia Appendix 1. MLM used a “self-supervised” algorithm that generated labels without human annotation. A note was first tokenized into a sequence of word-tokens, and 15% of the tokens were randomly selected. Among each selected token, there was an 80% probability it would be masked (replaced by a “[MASK]” token), a 10% probability it would be replaced by a random token, and a 10% probability it remains unchanged. The MLM pretraining trained the NLP model to do “cloze,” that is, input a sequence of word-tokens with masked tokens and predict the masked tokens using the context. It is hypothesized that through learning the cloze task, the NLP model can generalize this knowledge to improve the performance of other NLP tasks. We continuously pretrained the ClinicalBERT model with 74.0k head CT imaging notes from 2007 to 2020, including a total of 13.7 million words for 5 rounds (“epochs”), and used stand-alone 8.2k notes from January to July 2021 for MLM evaluation (Table S3 in Multimedia Appendix 1). This pretraining process produced a BERT model, which we labeled “HeadCT_BERT,” that is specific to the head CT imaging domain and can be further fine-tuned for downstream NLP tasks.

#### Fine-Tuning

To train the HeadCT_BERT for stroke features extraction, our downstream task in this study, we fine-tuned it with a development set of 200 notes annotated with stroke features. The HeadCT_BERT was appended with a feedforward layer with sigmoid activation function (“classification layer”) for the stroke feature classification. For each input segment (coded as a sequence of word-tokens with a maximum length of 64), the network outputs an array of probabilities (one probability for each stroke feature). The entire network (HeadCT_BERT + classification layer) was trained simultaneously. To prevent the model from becoming too attuned to the details of the development set, and consequently losing flexibility for new data (ie, to avoid overfitting), the development set was divided into a training set (80% of the notes) and a validation set (the remaining 20% of notes) [26]. Model weights were saved as checkpoints after each epoch, and optimal checkpoint weights were selected during validation as our final NLP model. The same fine-tuning process was also performed on the out-of-box ClinicalBERT model for comparison. The deep learning model was implemented using Python 3.9.6, PyTorch 1.9.0, and Transformers 4.10.0. Model computations were performed on an NVIDIA RTX 5000 graphics processing unit.

### Prediction and Evaluation

The NLP model predicts the probabilities of stroke features in each segment. We aggregated the prediction to note level by selecting the maximum probability of each stroke feature among segments. The final prediction for each note consists of a probability per stroke feature (multilabel classification). We considered stroke features with a probability >.5 as presence.

To evaluate our NLP model performance, we used a stand-alone evaluation set of 200 annotated imaging notes. Evaluation metrics included recall (sensitivity), specificity, precision (positive predictive value), and *F*_1_-score (the harmonic mean of precision and recall). *F*_1_-score ranges from 0 to 1, with 1 implying perfect model performance, AUROC curve, and accuracy. We also calculated predicted probabilities and fraction of stroke features and presented probability calibration curves (reliability diagrams).




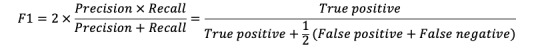




### Sensitivity Analysis

One challenge for NLP modeling is the need for a large amount of human annotation, which is time consuming and labor intensive. To explore the relationship between the number of annotated training notes and model performance, and potentially reduce the annotation workload, we performed a sensitivity analysis that compared NLP models that were fine-tuned with different development set sizes: 25, 50, 100, and 150 notes. Each subset was split into a training set (80%) and a validation set (20%) and was evaluated on the set of 200 notes.

### Structured Data Generation

Upon achieving satisfactory evaluation, we ran the model on all head CT imaging notes to automatically generate a structured data set of stroke imaging features. Each feature was represented as a binary variable (yes/no) associated with an imaging note. We further performed survival analysis with the Kaplan-Meier curves to evaluate the association between having any of the severe stroke features (eg, midline shift, perihematomal edema, and mass effect), as captured by NLP, and mortality for patients with acute ischemic stroke (AIS) and ICH. Differences in survival curves were compared using log-rank tests. We calculated survival rates and median survival days.

## Results

Of the 982,536 available images in REINAH, we identified 82,073 head CT imaging notes representing 24,924 unique patients, of whom, 13,439 (53.9%) were female, 14,028 (56.3%) were non-Hispanic White, and 15,121 (60.7%) were Medicare beneficiaries, with an overall median age of 69 (IQR 58.5-78.3) years. With regard to stroke subtypes (at the initial encounter), 12,623 (54.4%) of patients had AIS diagnosis, 1307 (5.6%) had subarachnoid hemorrhage (SAH), 7084 (30.5%) had a transient ischemic attack (TIA), and 2208 (9.5%) had ICH. For patients with AIS, the median National Institutes of Health Stroke Scale within 6 and 12 hours of admission was 3.0 (IQR 1.0-7.0), whereas it was 7.0 (IQR 2.0, 19.0) for patients with ICH. The 400 randomly sampled notes represented 398 unique patients. Their sociodemographic characteristics were consistent with the overall population of patients with head CT images. However, a greater proportion of sampled (vs full cohort) patients had a subarachnoid hemorrhage or an ICH, perhaps owing to head CT being a gold standard for evaluation of ICH. Although median BMI was not significantly different in the annotation sample (vs full cohort), the full cohort had a significantly higher proportion of missing BMI information (Table 2).

After annotation, stroke imaging features, including hemorrhage volume, herniation, ICH location, location of other relevant findings, remote stroke, subdural hematoma, and extra-axial hemorrhage, were excluded from modeling due to low frequencies (Table S1 in Multimedia Appendix 1). The interreviewer agreement analysis showed an excellent agreement between the 2 annotators (0.85 % average Kappa and 97.1% agreement).

Our fine-tuned HeadCT_BERT model had an AUROC of 0.9831 and an *F*_1_-score of 0.8683. The *F*_1_-scores were greater than 0.9 for 8 of 13 (61.5%) stroke imaging features, and the AUROCs were greater than 0.96 for all features except for acute ischemia. Results show that after fine-tuning, both ClinicalBERT and HeadCT_BERT achieved favorable performances, while HeadCT_BERT demonstrated marginally better performance (Table 3 and Table 4; Figure S2 in Multimedia Appendix 1).

The sensitivity analysis revealed sigmoid shapes for both models, indicating that improvement in model performance wanes as sample size approaches an optimal point. Specifically, we found marked performance improvements when increasing the training sample size from 25 to 50 and 100 notes. From 100 to 150, however, performance gain decreases, and from 150 to 200 notes, the performance gain is minimal, indicating that the NLP models had achieved near-optimal performance (Figure S1 in Multimedia Appendix 1).

The probability calibration curves showed HeadCT_BERT is well calibrated for some stroke features (eg, midline shift, white matter hyperintensity, subacute infarct, acute ischemia, subarachnoid hemorrhage, and encephalomalacia), while ClinicalBERT is well calibrated for midline shift, white matter hyperintensity, old stroke, subacute infarct, cerebral atrophy, acute ischemia, ICH, encephalomalacia, and mass effect (Figure S3 in Multimedia Appendix 1).

Running on a single–graphics processing unit server, our final NLP model processed ~230 imaging notes per minute and automatically generated a structured stroke imaging feature data set from 24,924 patients with head CT notes across the hospital system. In the resulting data set, 3826 (15.4%) of patients had a mass effect, 3600 (14.4%) had perihematomal edema, 1908 (7.7%) had a midline shift, and 5146 (20.6%) had 1 or more than 1 severe stroke features (eg, midline shift, mass effect, or perihematomal edema; Table 5).

**Figure 2:**
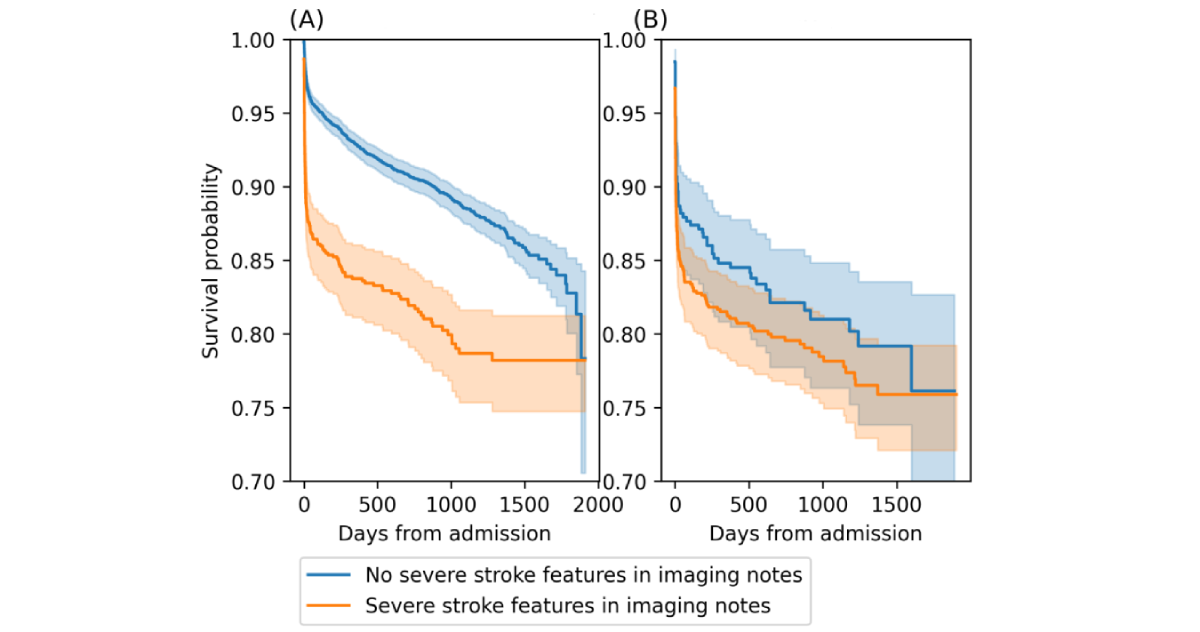
Kaplan-Meier curve of survival probability from initial admissions. Patients whose initial imaging includes severe stroke features (eg, midline shift, mass effect, or perihematomal edema) had a lower survival probability. (A) Acute ischemic stroke admissions (*P*<.001). (B) Intracerebral hemorrhage admissions (*P*=.19).

Survival analysis based on the initial head CT notes of 6463 AIS and 1243 ICH emergency admissions showed that patients with severe stroke features had higher mortality and shorter survival times (AIS: 18.4% mortality rate and 585 days median survival time; ICH: 20.7% mortality rate and 572 days median survival time) compared to other patients (AIS: 10.1% mortality rate and 759 days median survival time; ICH: 17.8% mortality rate and 638 days median survival time). Differences in survival probability over time are shown as Kaplan-Meier curves. Among AIS admissions, patients with severe stroke features had significantly lower survival probabilities (*P*<.001; Figure 2).

**Table 2:** Patient characteristics (average age and BMI are reported at imaging encounters). Italicized *P* values are significant.

Characteristics				Head CT^a^ population		Annotation sample		*P* value	
Imaging notes, N				82,073		400			
Unique patients, N				24,924		398			
Age (years), median (Q1, Q3)				69.0 (58.5, 78.3)		68.0 (56.4, 78.1)		.22	
**Age (years), n (%)**									.41
	0-49			3025 (12.1)		57 (14.3)			
	50-59			3793 (15.2)		61 (15.3)			
	60-69			6149 (24.7)		103 (25.9)			
	≥70			11,957 (48)		177 (44.5)			
**Gender, n (%)**									.69
	Female			13,439 (53.9)		219 (55)			
	Male			11,485 (46.1)		179 (45)			
**Race or ethnicity, n (%)**									.22
	Non-Hispanic White			14,028 (56.3)		206 (51.8)			
	Black			5690 (22.8)		102 (25.6)			
	Hispanic			3412 (13.7)		61 (15.3)			
	Asian			1209 (4.9)		16 (4)			
	Other or unknown			585 (2.3)		13 (3.3)			
BMI (kg/m^2^), median (Q1, Q3)				27.3 (23.7, 31.7)		27.3 (23.5, 31.0)		.59	
**BMI (kg/m^2^), n (%)**									*.001*
	Underweight			637 (2.6)		13 (3.3)			
	Normal			6193 (24.8)		108 (27.1)			
	Overweight			6518 (26.2)		123 (30.9)			
	Obese			6610 (26.5)		107 (26.9)			
	Missing			4966 (19.9)		47 (11.8)			
**Insurance^b^, n (%)**									
	**Medicare**								.15
		No	9803 (39.3)		142 (35.7)				
		Yes	15,121 (60.7)		256 (64.3)				
	**Medicaid**								.12
		No	23,793 (95.5)		373 (93.7)				
		Yes	1131 (4.5)		25 (6.3)				
	**Commercial**								*.04*
		No	20,194 (81)		306 (76.9)				
		Yes	4730 (19)		92 (23.1)				
	**Exchange**								.79
		No	24,437 (98)		389 (97.7)				
		Yes	487 (2)		9 (2.3)				
**Primary stroke type^c^, n (%)**									*<.001*
	Subarachnoid hemorrhage			1307 (5.6)		29 (7.7)			
	Transient ischemic attack			7084 (30.5)		100 (26.5)			
	Intracerebral hemorrhage			2208 (9.5)		59 (15.6)			
	Acute ischemic stroke			12,623 (54.4)		189 (50.1)			
**NIHSS^d^ Stroke Scale for acute ischemic stroke, median (Q1, Q3)**									
	Average NIHSS in 6 hours			3.0 (1.0, 7.0)		3.0 (1.5, 9.0)		.09	
	Average NIHSS in 12 hours			3.0 (1.0, 7.0)		3.0 (1.0, 8.0)		.24	
**NIHSS Stroke Scale for intracerebral hemorrhage, median (Q1, Q3)**									
	Average NIHSS in 6 hours			7.0 (2.0, 19.0)		6 (1.5, 18.0)		.94	
	Average NIHSS in 12 hours			7.0 (2.0, 19.0)		7.0 (2.0, 18.0)		.81	

^a^CT: computed tomography.

^b^Insurance type was collected throughout all imaging encounters.

^c^For patients with multiple stroke visits, the initial encounter’s stroke scale and primary stroke type are presented. We perform hypothesis testing to compare the 398 sampled patients with the nonsampled population. Chi-square tests were adopted for categorical variables, and Kruskal-Wallis tests were adopted for continuous variables.

^d^NIHSS: National Institutes of Health Stroke Scale.

**Table 3:** Final natural language processing model evaluation with the evaluation set (N=200) at the imaging note level.

Stroke feature	Specificity	Precision	Recall	*F*_1_-score	AUROC^a^ (95% CI)	Accuracy (95% CI)
Midline shift	1	1	0.9375	0.9677	0.9973 (0.9792-1.0154)	0.9950 (0.9852-1.0048)
Perihematomal edema	0.9945	0.9474	0.9474	0.9474	0.9994 (0.9917-1.0071)	0.9900 (0.9762-1.0038)
White matter hyperintensity	0.9725	0.9667	0.956	0.9613	0.9704 (0.9452-0.9955)	0.9650 (0.9395-0.9905)
Lacunes	1	1	1	1	1.0000 (1.0000-1.0000)	1.0000 (1.0000-1.0000)
Old stroke	0.9581	0.8056	0.8788	0.8406	0.9693 (0.9277-1.0110)	0.9450 (0.9134-0.9766)
Subacute infarct	0.9945	0.9091	0.5556	0.6897	0.9789 (0.9321-1.0258)	0.9550 (0.9263-0.9837)
Cerebral atrophy	0.9173	0.8571	0.9851	0.9167	0.9673 (0.9369-0.9978)	0.9400 (0.9071-0.9729)
Intraventricular hemorrhage	0.984	0.7273	0.6154	0.6667	0.9798 (0.9259-1.0338)	0.9600 (0.9328-0.9872)
Acute ischemia	0.956	0.6364	0.7778	0.7	0.9362 (0.8570-1.0154)	0.9400 (0.9071-0.9729)
Intracerebral hemorrhage	0.9665	0.75	0.8571	0.8	0.9872 (0.9532-1.0212)	0.9550 (0.9263-0.9837)
Subarachnoid hemorrhage	1	1	0.8333	0.9091	1.0000 (1.0000-1.0000)	0.9900 (0.9762-1.0038)
Encephalomalacia	1	1	0.9524	0.9756	0.9989 (0.9890-1.0088)	0.9950 (0.9852-1.0048)
Mass effect	0.9777	0.84	1	0.913	0.9952 (0.9743-1.0161)	0.9800 (0.9606-0.9994)

^a^AUROC: area under receiver operating characteristic curve.

**Table 4:** Average natural language processing model evaluation metrics among 13 stroke features for the fine-tuned models.

Stroke feature	*F*_1_-score, mean (SD)	AUROC^a^, mean (SD)	Accuracy, mean (SD)
HeadCT_BERT (final model)	*0.8683 (0.1176)^b^*	*0.9831 (0.0189)^b^*	*0.9700 (0.0225)^b^*
ClinicalBERT (baseline model)	0.8564 (0.1173)	0.9786 (0.0216)	0.9665 (0.0237)

^a^AUROC: area under receiver operating characteristic curve.

^b^Italicized values denote performance of the proposed model.

**Table 5:** Natural language processing (NLP) model generating structured stroke feature data sets from imaging notes^a^.

Characteristics	Head CT^b^ imaging patients^c^ (N=24924), n (%)	Acute ischemic stroke admission initial CT^d^ (N=6463), n (%)	Intracerebral hemorrhage admission initial CT^e^ (N=1243), n (%)
White matter hyperintensity	16,014 (64.3)	3429 (53.1)	407 (32.7)
Cerebral atrophy	13,615 (54.6)	2262 (35)	268 (21.6)
Old stroke	7426 (29.8)	1324 (20.5)	91 (7.3)
Lacunes	6622 (26.6)	1386 (21.4)	116 (9.3)
Mass effect	3826 (15.4)	614 (9.5)	500 (40.2)
Intracerebral hemorrhage	3822 (15.3)	354 (5.5)	1096 (88.2)
Perihematomal edema	3600 (14.4)	436 (6.7)	623 (50.1)
Encephalomalacia	3453 (13.9)	373 (5.8)	50 (4)
Acute ischemia	3426 (13.7)	1173 (18.1)	33 (2.7)
Subacute infarct	2675 (10.7)	841 (13)	28 (2.3)
subarachnoid hemorrhage	2179 (8.7)	132 (2)	245 (19.7)
Midline shift	1908 (7.7)	184 (2.8)	345 (27.8)
Intraventricular hemorrhage	1409 (5.7)	37 (0.6)	405 (32.6)
Severe stroke features^f^	5146 (20.6)	901 (13.9)	845 (68)

^a^Our final NLP model processed 82,073 head computed tomography notes for 24,924 unique patients in the entire hospital system and generated structured data sets.

^b^CT: computed tomography.

^c^The stroke features in the overall population were aggregated at the patient level.

^d,e^The stroke features in the initial head CT of acute ischemic stroke and intracerebral hemorrhage emergency admissions were presented.

^f^Severe stroke features include midline shift, perihematomal edema, or mass effect. Severe stroke feature is a composite feature.

## Discussion

### Principal Findings

We propose an NLP pipeline to extract ischemic and hemorrhagic stroke characteristics from head CT imaging notes (HeadCT_BERT model). Built upon one of the latest clinical NLP models, the HeadCT_BERT model achieved an excellent average AUROC of 0.9831 and an accuracy of 97%. Our NLP pipeline showed promising performance for the detection of midline shift, perihematomal edema, lacunes, subarachnoid hemorrhage, encephalomalacia, and mass effect, with AUROCs for each of these features exceeding 0.99 and *F*_1_-scores above 0.9 for the evaluation set. Other features, including white matter hyperintensity, old stroke, subacute infarct, cerebral atrophy, intraventricular hemorrhage, and ICH showed AUROCs between 0.96 to 0.98. Other NLP studies have achieved optimal AUROC values of 0.9625 for mass effect extraction [17], 0.96 for stroke presence, and 0.93 for stroke acuity [1]. Our method achieved comparable or better performance for extracting stroke imaging features.

In 2018 alone, 11.5 million head CT scans were performed in the United States [27], generating valuable information that can be used to answer a multitude of stroke-related research questions. In the absence of methods to extract information in unstructured formats, the generation of insights from such sources is limited. This underscores the value of our NLP pipeline, which provides a fast, scalable, and automatic solution for the processing of unstructured text data.

Application of our pipeline in a health care environment has the potential to benefit both medical research and patient safety. For example, in this study, we demonstrated the use of NLP for retrospectively identifying cohorts of patients with AIS and ICH with severe stroke features. We identified 901 (13.9%) AIS and 845 (68%) patients with ICH with severe stroke neuroimaging features and demonstrated lower survival rates for patients with these severe features, consistent with previous studies [28,29]. Beyond outcome prediction, modifications of our pipeline may also be implemented to improve patient safety. For example, NLP pipelines that detect incidents can be used to improve patient outreach workflows by optimizing reporting procedures for health care providers as well as the patients and their families [30]. Our pipeline has the potential to process imaging notes in real time, generate flags for severe stroke findings, and trigger reminders and alerts within the EHR system.

Despite the performance of our NLP pipeline, this study has limitations. First, it was conducted and evaluated in a single organization, where many of the notes may have been written by a relatively small number of radiologists or neuroradiologists. Therefore, the generalizability of the trained NLP models could be limited by overly consistent wording and grammar in training data. However, as one of the largest hospital systems, comprising 7 certified stroke care hospitals in the Houston metropolitan area, we feel that our inclusion of a diverse collection of notes yields enough variability in the training data to mitigate this issue. Second, although our HeadCT_BERT model demonstrated slightly improved performance for stroke features extraction, it is hard to compare our model with ClinicalBERT due to the lack of well-established NLP benchmarks for head imaging reports. Future efforts to create head imaging NLP benchmarks are needed for comprehensive evaluation. Finally, the probability calibration curves of both HeadCT_BERT and ClinicalBERT for individual stroke features demonstrate a mixed performance in calibration, indicating potential imbalance of certain stroke features in the training data set. As a result, using a probability of .5 as a general cut-off might not be optimal for all stroke features. Future work is required to adequately calibrate the model for all stroke features.

### Conclusions

This study represents a step forward in NLP adoption for neuroimaging among patients with cerebrovascular disease. Our work demonstrates an effective and customizable NLP pipeline for retrieving multiple stroke features from large amounts of unstructured imaging notes. Derived from the latest artificial intelligence technology, we believe our model will benefit stroke research and patient safety. To fully understand the impact on the health care industry, future work in the data pipeline deployment and evaluation is anticipated.

## Appendix

Multimedia Appendix 1 Supplementary tables and figures.

## Abbreviations

AIS: acute ischemic stroke
AUROC: area under receiver operating characteristic curve
BERT: bidirectional encoder representations from transformers
CT: computed tomography
EHR: electronic health record
ICH: intracerebral hemorrhage
MLM: masked language model
MRI: magnetic resonance imaging
NLP: natural language processing
REINAH: Registry for Neurological Endpoint Assessments among Patients with Ischemic and Hemorrhagic Stroke
SVM: Support vector machine
