# Supplementary material for: Extraction of Radiological Characteristics From Free-Text Imaging Reports Using Natural Language Processing Among Patients With Ischemic and Hemorrhagic Stroke: Algorithm Development and Validation
Source: JMIR AI. 2023 Jun 6;2:e42884. doi: 10.2196/42884 (PMC11041442; doi:10.2196/42884)
Supplement: Multimedia Appendix 1 [file ai_v2i1e42884_app1.docx]

Table S1: Stroke concept frequency based on human annotation

| **Stroke feature** | **Involved in Notes** | **Included in the final model** |
| --- | --- | --- |
| **N (%)** | **400** |  |
| White matter hyperintensity | 167 (41.75) | Yes |
| Cerebral atrophy | 130 (32.5) | Yes |
| Old stroke | 70 (17.5) | Yes |
| Intracerebral Hemorrhage | 55 (13.75) | Yes |
| Lacunes | 53 (13.25) | Yes |
| Mass Effect | 49 (12.25) | Yes |
| Perihematomal Edema | 48 (12) | Yes |
| Midline shift | 37 (9.25) | Yes |
| Subacute Infarct | 36 (9) | Yes |
| Acute Ischemia | 36 (9) | Yes |
| Encephalomalacia | 35 (8.75) | Yes |
| Intraventricular hemorrhage | 30 (7.5) | Yes |
| subarachnoid hemorrhage | 23 (5.75) | Yes |
| ICH location | 14 (3.5) | No |
| Subdural Hematoma | 14 (3.5) | No |
| Herniation | 6 (1.5) | No |
| Extra-axial Hemorrhage | 5 (1.25) | No |
| Remote stroke | 1 (0.25) | No |
| Hemorrhage Volume | 0 (0) | No |
| Finding location | 0 (0) | No |

Figure S1: Sensitivity analysis shows the relationship between the development set size and model performances evaluated by F1 score, AUROC, and overall accuracy


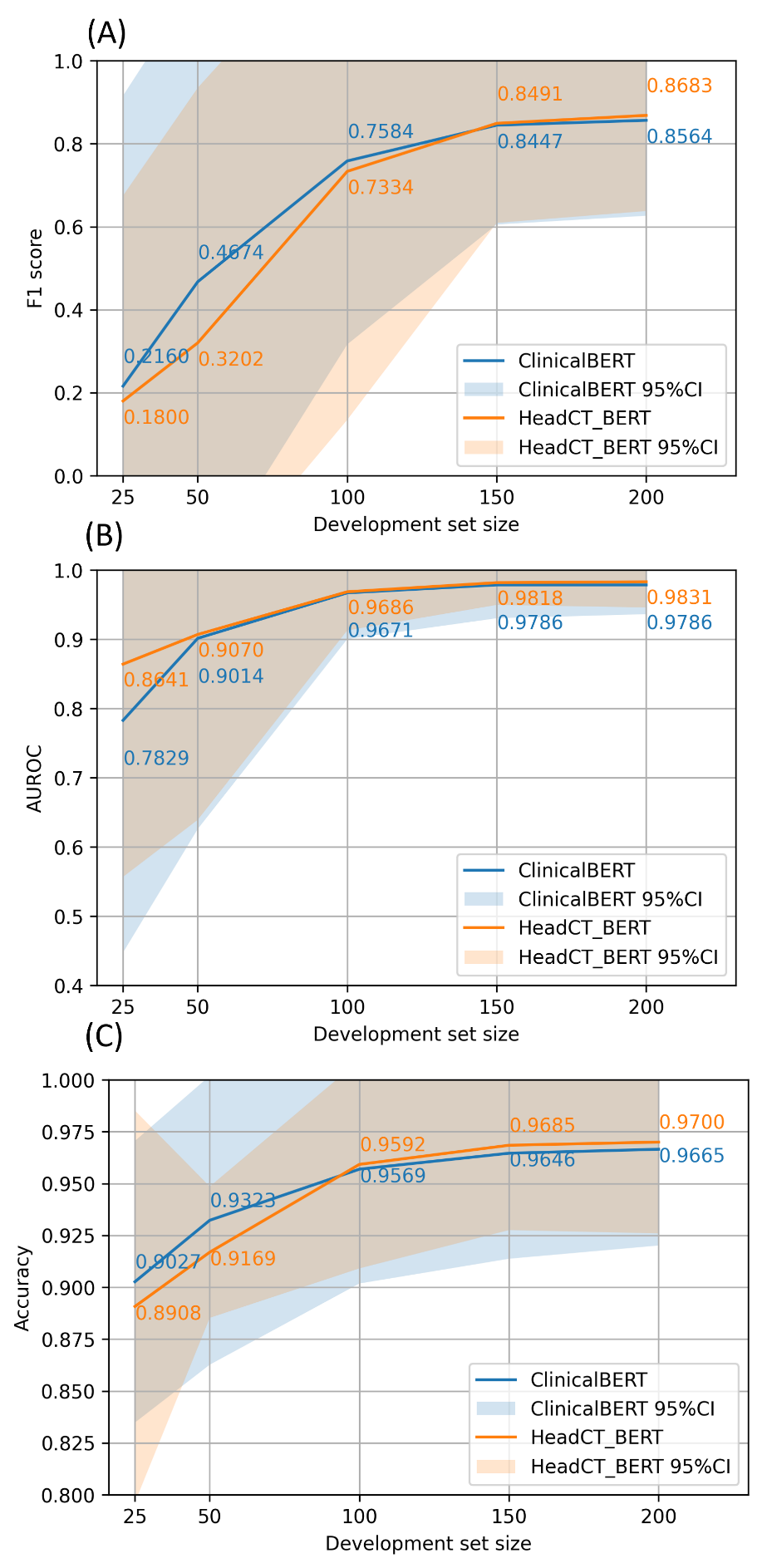


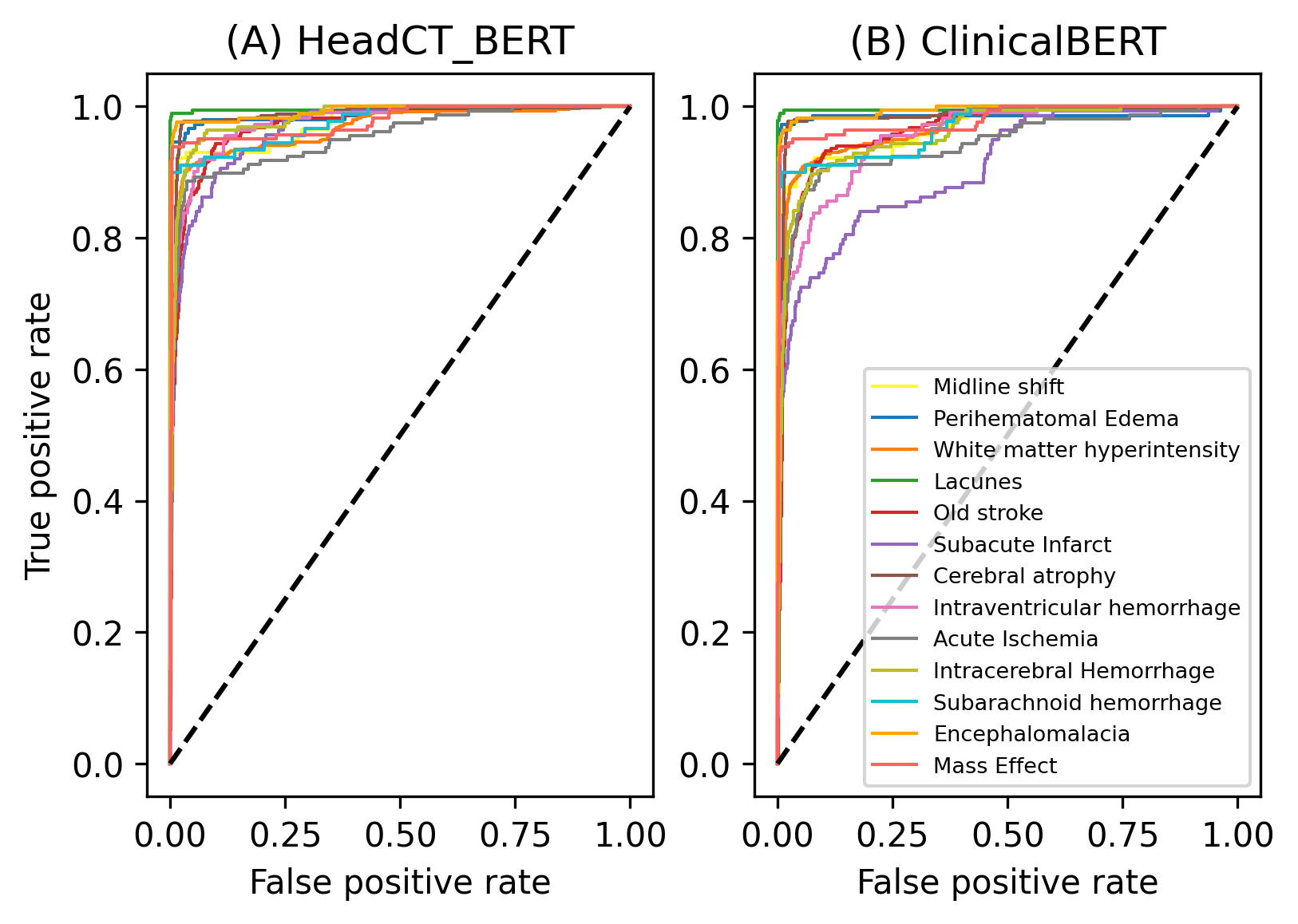


**Figure S2: Receiver Operating Characteristic (ROC) curves of the stroke features.** The NLP model predicts a probability of the presence of each stroke feature in a head CT imaging note (multi-label classification). (A) HeadCT_BERT showed a slightly improved performance over the (B) ClinicalBERT for stoke features like intraventricular hemorrhage and subacute infarct.


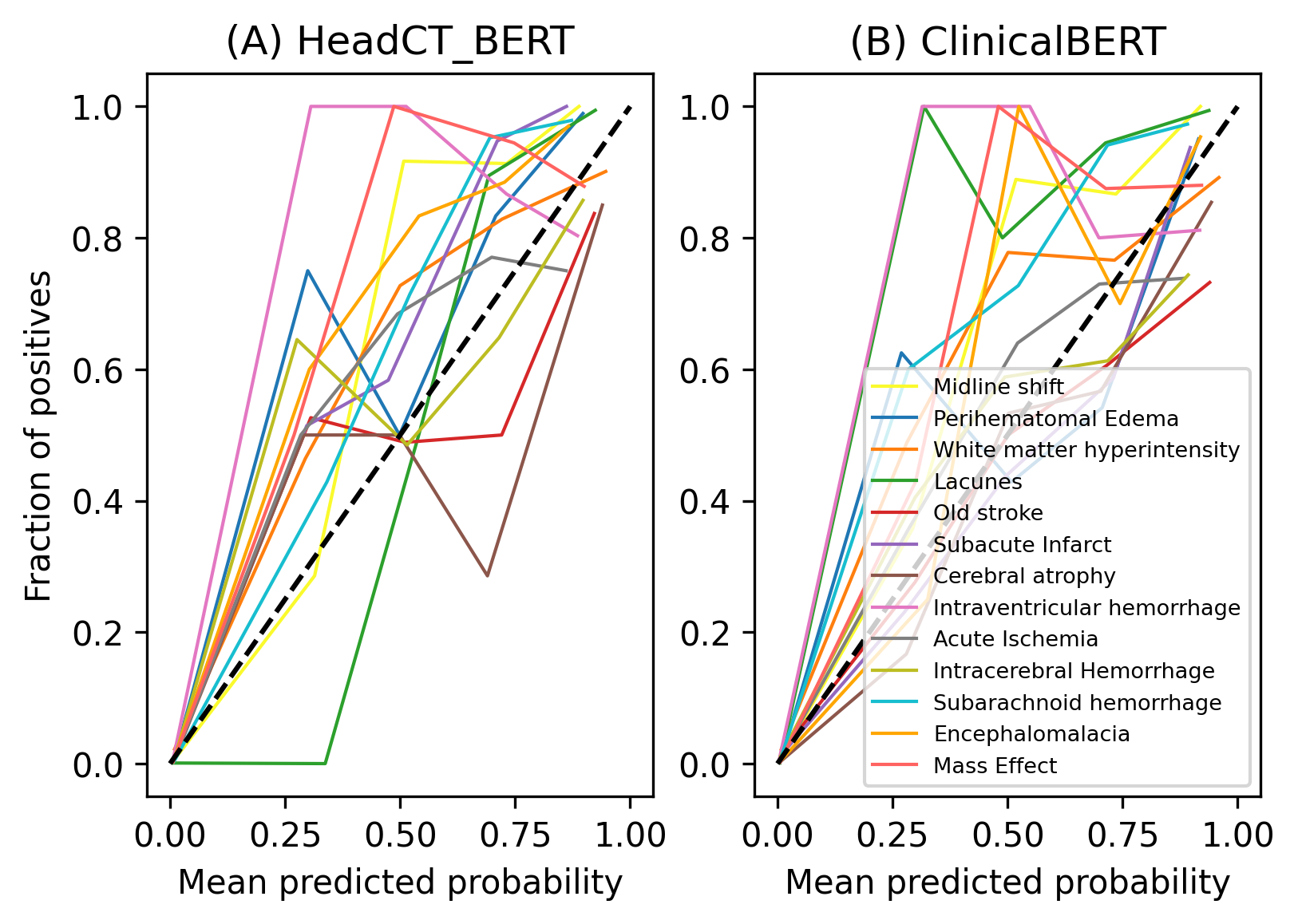


**Figure S3: Probability calibration curves of the stroke features.** (A) HeadCT_BERT is well calibrated on some stroke features (e.g., midline shift, white matter hyperintensity, subacute infarct, acute ischemia, subarachnoid hemorrhage, encephalomalacia), (B) ClinicalBERT is well calibrated on midline shift, white matter hyperintensity, old stroke, subacute infarct, cerebral atrophy, acute ischemia, intracerebral hemorrhage, encephalomalacia, and mass effect.

Table S2: Development of pre-trained NLP models

| **NLP models** | **Base NLP model** | **Training data** | **Reference** |
| --- | --- | --- | --- |
| BERT_base | From scratch | BooksCorpus (800M words) and English Wikipedia (2,500M words) | Devlin et al (2018)[12] |
| BioBERT | BERT base | PubMed Abstracts (4500M words) and PMC Full-text articles (13500M words) | Lee et al (2019)[25] |
| ClinicalBERT | BioBERT | MIMIC-III (880M words) | Alsentzer et al (2019)[13] |
| HeadCT_BERT | ClinicalBERT | Houston Methodist head CT imaging notes (13.7M words) | This manuscript |
| The process of the NLP model continuous pre-training went from the general domain to a specific field: The BERT_base model is pre-trained from scratch with BooksCorpus and English Wikipedia. The Bio-BERT uses BERT_base as the base model and is continuously pre-trained with PubMed abstracts and PMC articles. The ClinicalBERT uses BioBERT as the base model and is continuously pre-trained with MIMIC-III. Our proposed HeadCT_BERT used ClinicalBERT as the base model and was continuously pre-trained with Houston Methodist head CT imaging notes. | | | |

Table S3: NLP model pre-training and fine-tuning settings

| **Training set & Hyperparameters** | **Pre-training** | **Fine-tuning** |
| --- | --- | --- |
| Base NLP model | *ClinicalBERT* | *HeadCT_BERT* |
| Development set | 74K unlabeled head CT imaging notes from 2007 to 2020 | 200 annotated notes in training set |
| Evaluation set | 8K unlabeled head CT imaging notes from January to July 2021 | 200 annotated notes in evaluation set |
| Optimizer | Adam | Adam |
| Learning rate | 2e-5 | 2e-5 |
| Batch size | 32 | 32 |
| Input length (tokens) | 64 | 64 |
| Epochs | 5 | 9 |
| Steps | 27.4k | 1.1k |
| Product | A head CT domain-specific base NLP model “*HeadCT_BERT*” ready to be fine-tuned for downstream tasks | A fine-tuned NLP model for extracting stroke features |
| We used *ClinicalBERT* as the base model and continuously pre-trained it with 74K unlabeled head CT imaging notes. The product was a domain-specific NLP model (“*HeadCT_BERT*”) which can be fine-tuned to solve downstream NLP tasks. We then fine-tuned *HeadCT_BERT* with 200 annotated notes for stroke feature extraction.  **MLM evaluation**: The *HeadCT_BERT* achieved an 0.9243 (0.9227-0.9253) accuracy in predicting the 27,255 masked word-tokens in imaging notes from January to July 2021 while the out-of-box *ClinicalBERT* had 0.5973 (0.5950-0.5996) accuracy. Note that the performance on MLM does not always correlate with the performance on downstream tasks. Evaluation of each downstream task is more meaningful. | | |
